# Development of skeletal muscle atrophy and intermuscular adipose tissue in patients with early breast cancer treated with chemotherapy

**DOI:** 10.1152/ajpcell.00373.2022

**Published:** 2022-09-12

**Authors:** Joris Mallard, Elyse Hucteau, Laura Bender, Anouk Charlot, Léa Debrut, Carole Pflumio, Philippe Trensz, Roland Schott, Fabrice Favret, Xavier Pivot, Thomas J. Hureau, Allan F. Pagano

**Affiliations:** ^1^Faculté de médecine, maïeutique et sciences de la santé, “Mitochondrie, Stress oxydant, Protection musculaire,” Université de Strasbourg, Strasbourg, France; ^2^Faculté des Sciences du Sport, Centre Européen d’Enseignement de Recherche et d’Innovation en Physiologie de l’Exercice (CEERIPE), Université de Strasbourg, Strasbourg, France; ^3^Institut de Cancérologie Strasbourg Europe (ICANS), Strasbourg, France

**Keywords:** fatty infiltrations, fibro-adipogenic progenitors (FAPs), intermuscular adipose tissue (IMAT), muscle architecture, skeletal muscle deconditioning

## Abstract

Chemotherapy is a common therapy to treat patients with breast cancer but also leads to skeletal muscle deconditioning. Skeletal muscle deconditioning is multifactorial and intermuscular adipose tissue (IMAT) accumulation is closely linked to muscle dysfunction. To date, there is no clinical study available investigating IMAT development through a longitudinal protocol and the underlying mechanisms remain unknown. Our study was dedicated to investigating IMAT content in patients with early breast cancer who were treated with chemotherapy and exploring the subsequent cellular mechanisms involved in its development. We included 13 women undergoing chemotherapy. Muscle biopsies and ultrasonography assessment were performed before and after chemotherapy completion. Histological and Western blotting analyses were conducted. We found a substantial increase in protein levels of three mature adipocyte markers (perilipin, +901%; adiponectin, +135%; FABP4, +321%; *P* < 0.05). These results were supported by an increase in oil red O-positive staining (+358%; *P* < 0.05). A substantial increase in PDGFRα protein levels was observed (+476%; *P* < 0.05) highlighting an increase in fibro-adipogenic progenitors (FAPs) content. The cross-sectional area of the vastus lateralis muscle fibers substantially decreased (−21%; *P* < 0.01), and muscle architecture was altered, as shown by a decrease in fascicle length (−15%; *P* < 0.05) and a decreasing trend in muscle thickness (−8%; *P* = 0.08). We demonstrated both IMAT development and muscle atrophy in patients with breast cancer who were treated with chemotherapy. FAPs, critical stem cells inducing both IMAT development and skeletal muscle atrophy, also increased, suggesting that FAPs likely play a critical role in the skeletal muscle deconditioning observed in patients with breast cancer who were treated with chemotherapy.

## Graphical abstract

**Figure:**
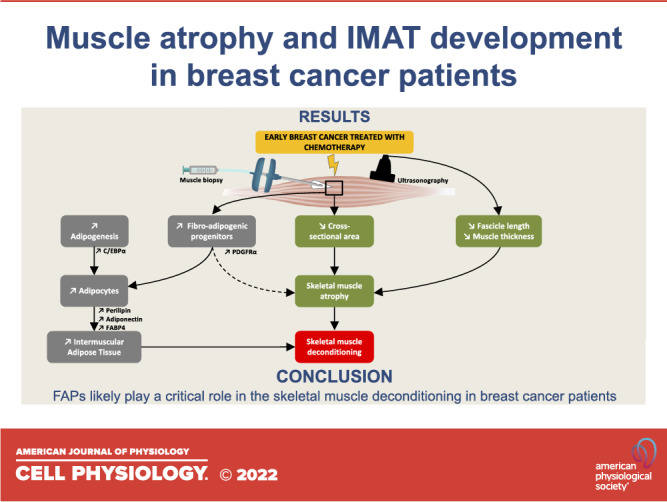

## INTRODUCTION

Chemotherapy is a common therapy to treat patients with early breast cancer that leads to severe off-target side effects that profoundly affect the patients’ quality of life and treatment tolerance. Among these side effects, skeletal muscle deconditioning is a significant adverse event in patients with breast cancer treated by chemotherapy and is characterized by a substantial decrease in muscle mass and force, ultimately leading to reduced exercise tolerance (1). If structural alterations such as a decrease in myofiber cross-sectional area (CSA) play a critical role in muscle atrophy, impairment in muscle quality drastically affects exercise tolerance (1, 2). In a previous study (3), we documented severe mitochondrial alterations in patients with breast cancer that may explain the decrease in cardiorespiratory fitness (4). Nevertheless, other factors should be studied, such as intermuscular adipose tissue (IMAT) development (1, 2, 5).

IMAT is characterized by the presence of adipocytes between muscle fibers and groups (2). An exacerbated accumulation is considered as a characteristic of skeletal muscle deconditioning, leading to a decrease in muscle force and exercise tolerance (2, 5, 6). Interestingly, a preclinical study reported that even if IMAT accumulation occurs concomitantly with muscle atrophy, it has an independent impact on muscle contraction (7). To date, two cross-sectional studies compared patients with breast cancer to healthy subjects and showed, through magnetic resonance imagery, an increase in thigh IMAT content. In a recent study conducted in patients with breast cancer, it was concluded that IMAT was a significant prognostic factor of survival outcomes, highlighting the importance of IMAT development in patients’ health evolution (8).

As reported in our recent review (1), we did not find any study exploring the cellular mechanisms involved in IMAT development in breast cancer. Previous studies in muscle disuse or pathological conditions consensually identified that fibro-adipogenic progenitors (FAPs) could differentiate into adipose and fibrotic tissue (9–11). FAPs represent a population of muscle-specific mesenchymal stem cells located in the interstitial space of muscle fibers (12). They are the primary cells expressing the cell surface receptor PDGFRα and trigger the development of IMAT (2, 13). In the context of patients with cancer, only one study reported that patients with cachectic pancreatic cancer displayed an increase in FAP content with exacerbated fibrotic tissue and lipids deposits (14).

To date, although patients with breast cancer experience increased IMAT content compared with healthy subjects (15, 16), there is no study investigating IMAT content through a longitudinal clinical study protocol. The underlying mechanisms remain unknown, and a more comprehensive understanding of the cellular processes is needed. Thus, our study was dedicated to investigating IMAT content in patients with early breast cancer who were treated with chemotherapy and exploring the subsequent cellular mechanisms involved in its development.

## MATERIAL AND METHODS

### Participants and Study Design

As fully described previously (3), 13 patients (56 ± 12 yr; 76 ± 15 kg) from the Institut de Cancérologie Strasbourg Europe (ICANS) were included in this study (NCT04638712). The eligibility criteria included nonpregnant women at least 18 yr old with early breast cancer treated with chemotherapy including an epirubicin-cyclophosphamide regimen followed by weekly paclitaxel. All patients provided written informed consent before enrollment, and the study was conducted in accordance with the Declaration of Helsinki and the ethics approval received from the national ethics committee (Registration Number 2020-A01266-33). Two visits were performed: before the first chemotherapy (CT) administration (pre-CT) and within 1 wk after the last CT administration (post-CT).

### Skeletal Muscle Biopsies

Skeletal muscle biopsies were obtained pre- and post-CT from the left vastus lateralis muscle using a 5-mm Bergström biopsy needle under local anesthesia (3). Tissue for histological analyses was embedded in small silicone casts filled with a cryoprotectant agent (OCT, Sakura, Finetek), immediately cooled in 2-methylbutane immersed in liquid nitrogen and stored at −80°C. Muscle tissue for Western blotting analysis was immediately frozen in liquid nitrogen and stored at −80°C.

### Cryosectioning and Histological Analyses

Transverse serial cross sections (7 μm) were obtained using a cryostat maintained at −20°C. Sections were then blocked (1× PBS/5% normal serum/0.3% Triton X-100), and laminin primary antibody (Thermo Fisher Scientific, MA1-06100, 1:100) was diluted in dilution buffer (1× PBS/1% BSA/0.3% Triton X-100) and incubated overnight at 4°C. Three washes with PBS were performed before applying secondary antibody (Abcam, ab175476, 1:1,000) at room temperature for 1 h, and ProLong diamond antifade mountant was used (Thermo Fisher Scientific, 36961). Stained slides were digitalized with the Zeiss Apotome.2 microscope with a ×20 objective (Hamamatsu), and myofiber CSA was semiautomatically quantified using SMASH application on MATLAB software (v. R2021b) (17). In addition, we investigated the lipid content through oil red O staining. Only lipids outside muscle fibers were quantified (MATLAB software v. R2021b) to exclude myocellular triglyceride content and then reflect IMAT deposits. Finally, collagen area was investigated through modified Masson’s trichrome staining (18) and quantified with MATLAB software (v. R2021b).

### Western Blotting and Antibodies

Procedures of Western blotting were fully described previously (3). Primary antibodies from Cell Signaling were used: anti-adiponectin (No. 2789, 1:500), anti-C/EBPα (No. 8178S, 1:1,000), anti-PDGFRα (No. 3174S, 1:1,000), anti-perilipin (No. 9349S, 1:1,000); from Thermo Fisher Scientific: anti-collagen I (PA5-95137, 1:1,000), anti-FAPB4 (No. 710189; 1:500), anti-fibronectin (PA5-29578, 1:1,000); and from Santa Cruz: anti-PPARγ (Sc-150, 1:200). Anti-rabbit (Cell Signaling, 1:4,000, No. 7074S) or anti-mouse (Cell Signaling, 1:4,000, No. 7076S) were used as secondary antibodies and protein ladder from Thermo Fisher Scientific was used (26620). Proteins were visualized by enhanced chemiluminescence (iBright 1500 Imaging System, Invitrogen) and quantified with ImageJ Software (v.1.8.0). Ponceau coloration was used as the loading control.

### Ultrasonography

The muscle architecture of the right vastus lateralis muscle was investigated using an ultrasound scanner (Toshiba Aplio XV; Toshiba Medical Systems, Tochigi, Japan) with a 58-mm linear probe (PLT-805AT 8.0-MHz). Patients were lying down, and their knees were extended and relaxed. A water-soluble transmission gel was applied to the scanning head of the probe, placed perpendicular to the skin and longitudinally at 62.5% between the anterior superior iliac spine and the lateral condyle of the femur. Muscle thickness and fascicle length (through extrapolation of the visible part of the fascicle) were calculated as previously described (19). Echogenicity was used as an indirect reflection of muscle quality and IMAT content (20) and was determined based on the grayscale analysis of each pixel contained in three squares with similar length. Five different images were analyzed by two independent investigators using ImageJ software (v.1.8.0) and the mean was calculated and used for statistical analyses.

### Sample Size Calculation and Statistical Analysis

As described previously (3), we conducted a sample size calculation and enrolled 13 patients. The Shapiro–Wilk test was used to test the normality of the data. Appropriate parametric or nonparametric analyses were then performed (*t* test or Wilcoxon tests, respectively) to compare pre- and post-CT variables. Statistical significance was set at *P* < 0.05, and all values are expressed as the means ± standard deviation (SD). Statistical analyses and graphs were made with GraphPad Prism 8 software.

## RESULTS

The data collected in this study were part of the same experimental protocol as Mallard et al. (3). There is no overlap in the data presented apart from patient characteristics.

### Patient Characteristics

We found no variation between the pre- and post-CT measurements for body weight (76 ± 15 kg vs. 75 ± 14 kg; *P* = 0.7). Two patients were unwilling to undergo the second muscle biopsy, and one patient declined the second ultrasonography procedure, resulting in *n* = 11 for muscle biopsy analyses and *n* = 12 for muscle architecture analyses.

### IMAT, Adipogenesis, and Fibro-Adipogenic Progenitor Content

**Figure 1.**
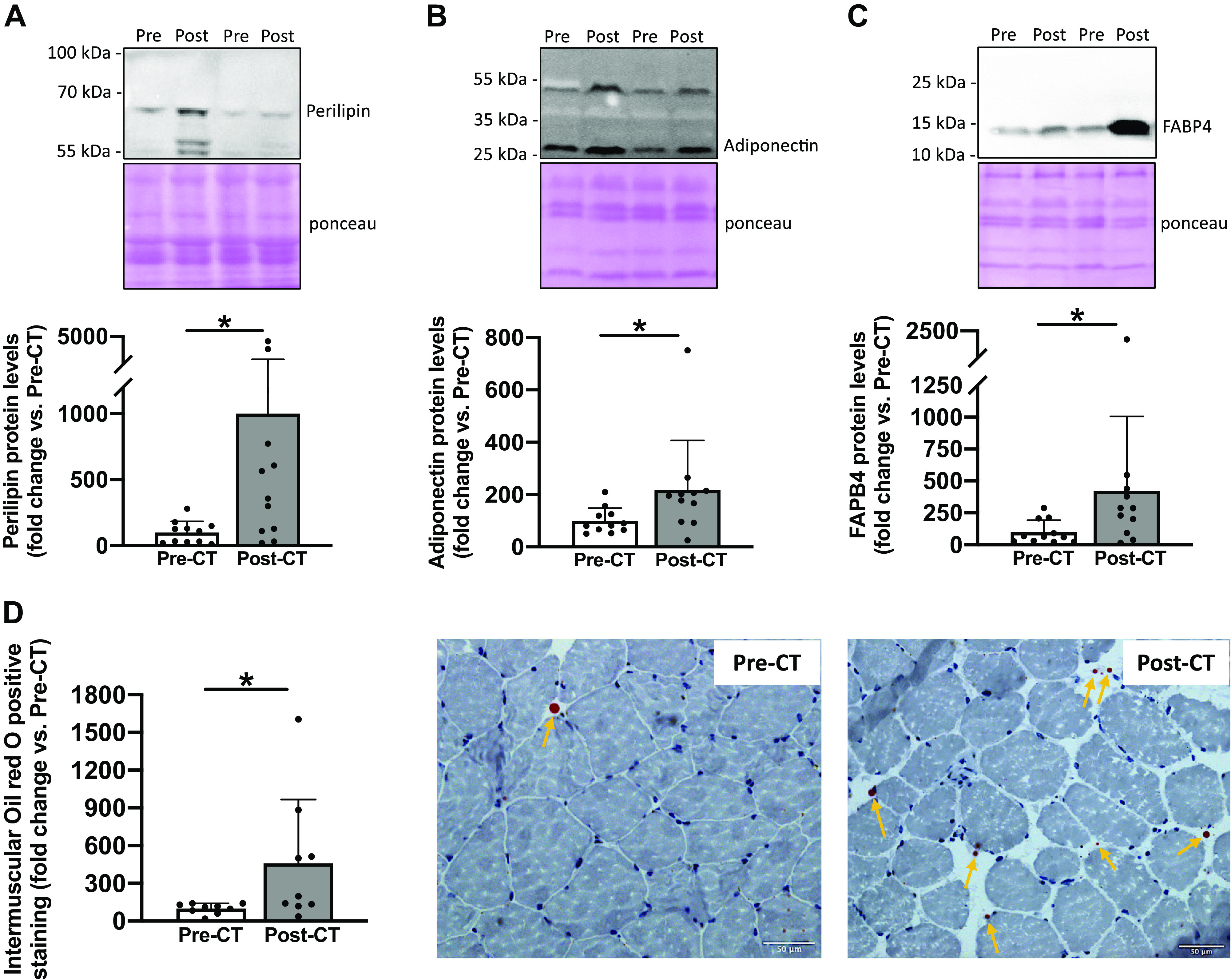
Changes in key markers of intermuscular adipose tissue content. Perilipin (*A*), adiponectin (*B*), and FABP4 (*C*) protein levels from vastus lateralis muscle biopsies taken before (Pre) and after (Post) chemotherapy (CT) (*n* = 11). For each Western blot, two representative subjects are displayed. Oil red O staining from transverse cross sections of vastus lateralis muscle biopsies pre- and post-CT and representative images of muscle transversal sections (*D*). Biopsy samples from two patients were of poor quality and were excluded from histological analysis (*n* = 9). All values are expressed as the means ± SD. *P < 0.05.

**Figure 2.**
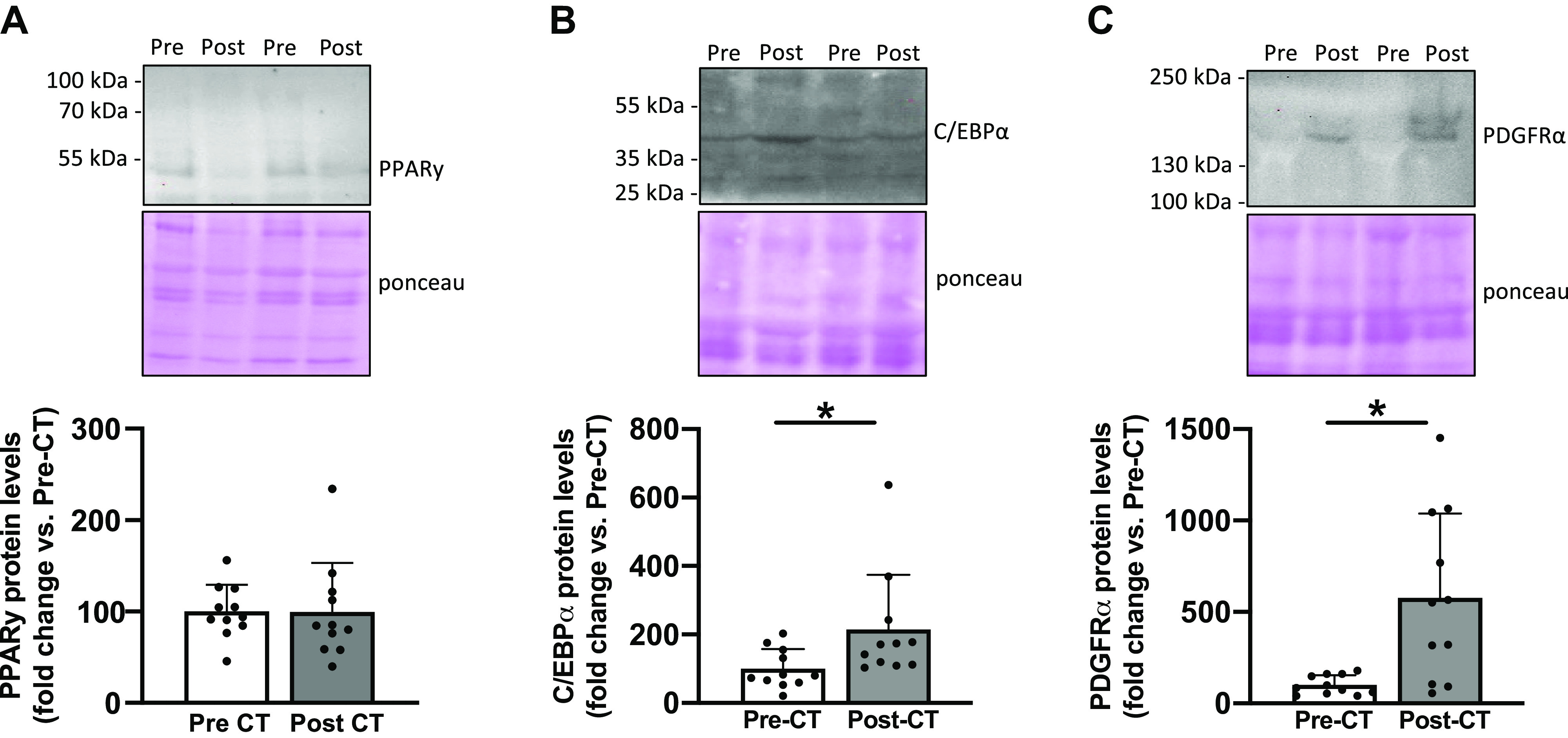
Changes in key markers of adipogenesis and fibro-adipogenic progenitor content. PPARγ (*A*), C/EBPα (*B*), and PDGFRα (*C*) protein levels from vastus lateralis muscle biopsies taken before (Pre) and after (Post) chemotherapy (CT) (*n* = 11). For each Western blots, two representative subjects are displayed. All values are expressed as the means ± SD. *P < 0.05.

We found a substantial increase in mature adipocyte markers post-CT, reaching +901% for perilipin protein levels (*P* < 0.05; Fig. 1*A*), +135% for adiponectin protein levels (*P* < 0.05; Fig. 1*B*), and +321% for FAPB4 protein levels (*P* < 0.05; Fig. 1*C*). These results were supported by an increase in oil red O-positive staining quantified only between the muscle fibers (+358%; *P* < 0.05, Fig. 1*D*). Altogether, these results highlighted an increase in IMAT content. We therefore investigated late adipogenic transcription factors, and despite no change in PPARγ protein levels (*P* = 0.6; Fig. 2*A*), an increase in C/EBPα protein levels was found (+114%; *P* < 0.05; Fig. 2*B*). Finally, a substantial increase in PDGFRα protein levels was observed post-CT, reaching +476% (*P* < 0.05; Fig. 2*C*) and highlighting an increase in FAP content following CT.

### Fibrosis Markers Expression

**Figure 3.**
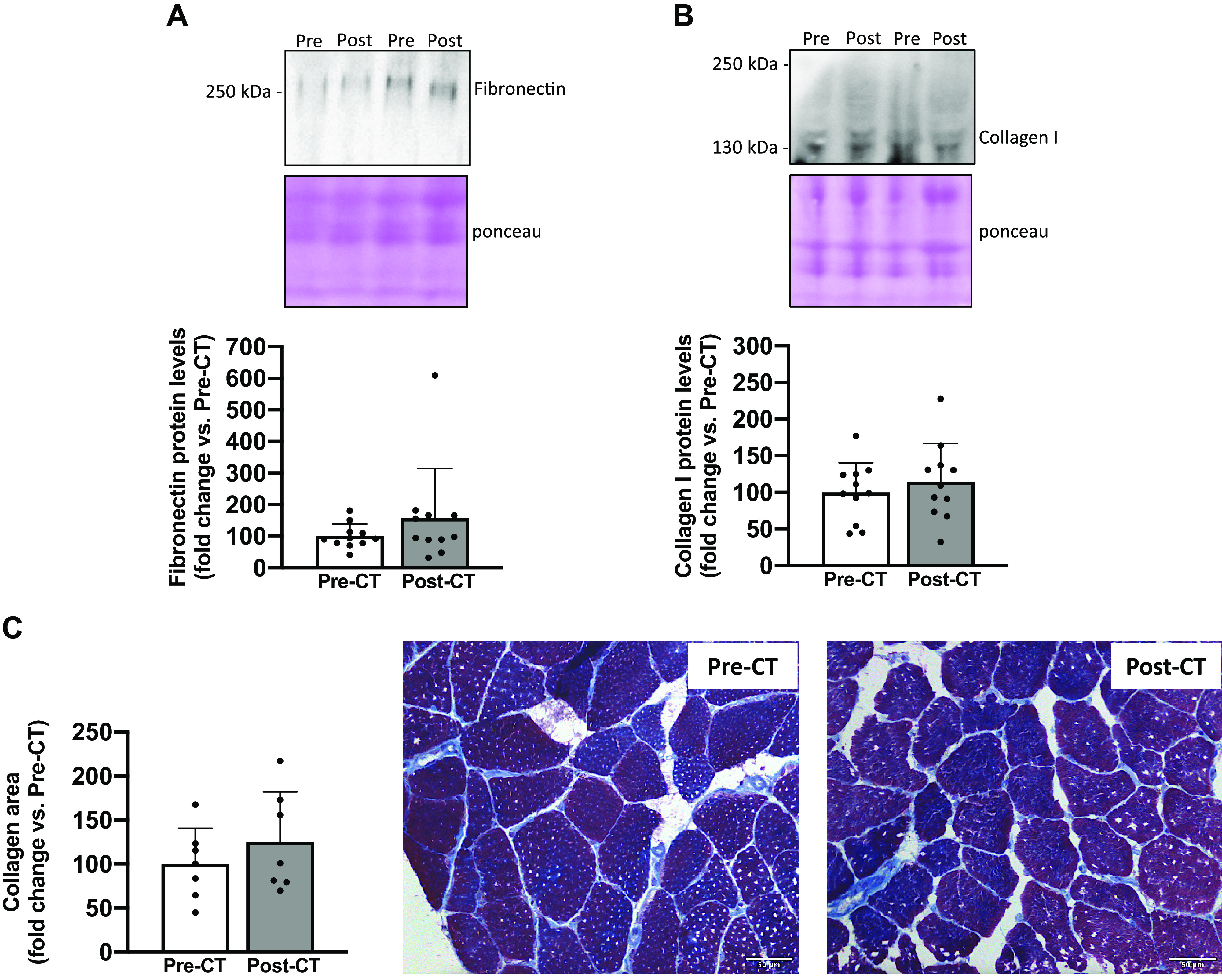
Changes in markers of fibrosis. Fibronectin (*A*) and collagen I (*B*) protein levels from vastus lateralis muscle biopsies taken before (Pre) and after (Post) chemotherapy (CT) (*n* = 11). For each Western blot, two representative subjects are displayed. Modified Masson’s trichrome staining from transverse cross sections of vastus lateralis muscle biopsies pre- and post-CT and representative images of muscle transversal sections with collagen stained in blue (*C*). Biopsy samples from four patients were of poor quality and were excluded from histological analysis (*n* = 7). All values are expressed as the means ± SD. *P < 0.05.

Considering that FAPs are also able to differentiate into fibrotic tissue, we investigated key fibrosis markers. We did not report any changes in fibronectin protein levels (*P* = 0.6; Fig. 3*A*) or collagen I protein levels (*P* = 0.5; Fig. 3*B*). These results were further supported by the absence of change in collagen area (*P* = 0.5; Fig. 3*C*).

### Muscle Atrophy, Architecture, and Echogenicity

**Figure 4.**
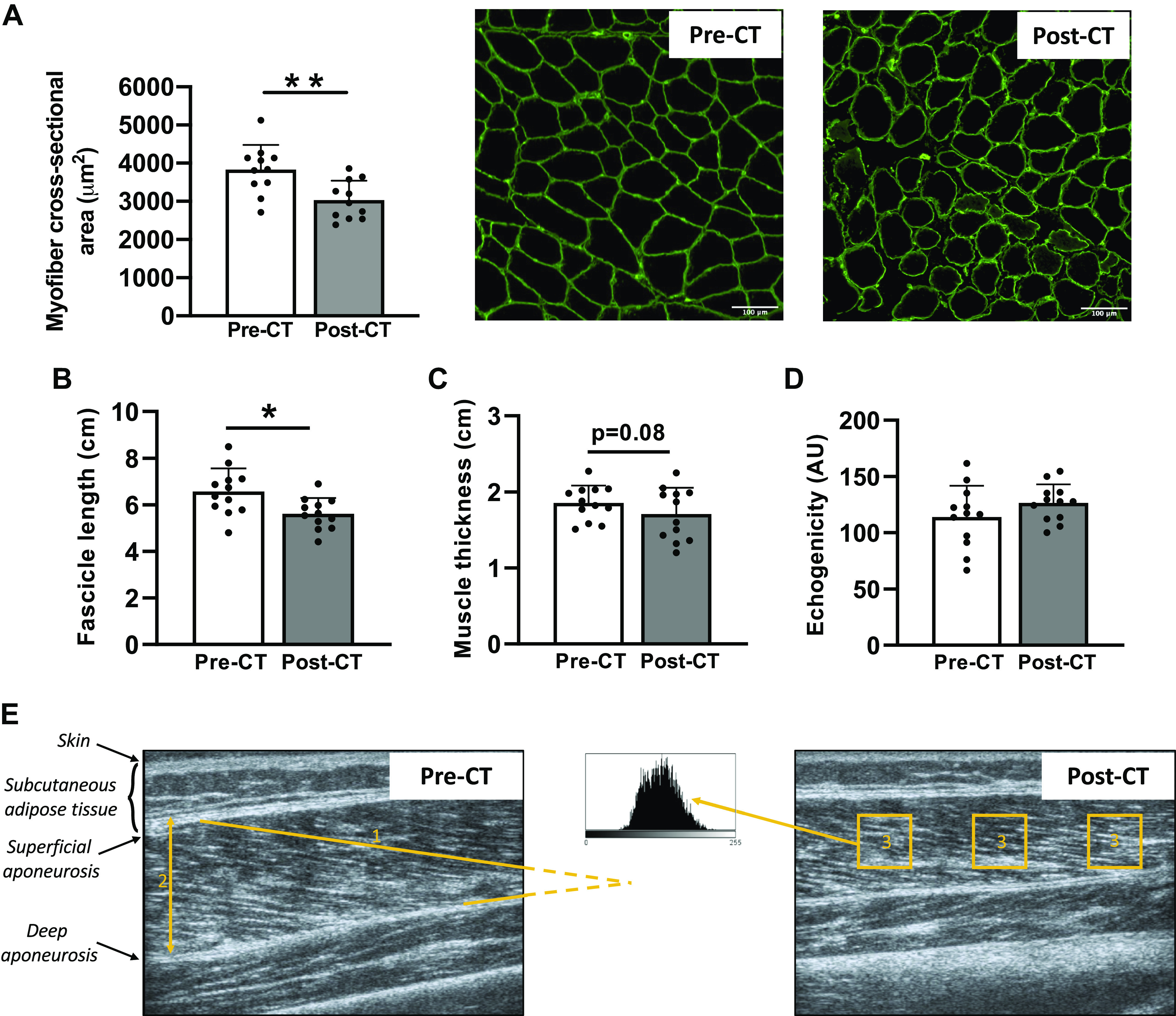
Changes in cross-sectional area, muscle architecture, and echogenicity of the vastus lateralis muscle. Cross-sectional area of vastus lateralis muscle fibers (*A*) before (Pre) and after (Post) chemotherapy (CT) and representative transversal muscle sections with laminin staining (*n* = 11). Fascicle length (*B*), muscle thickness (*C*), and echogenicity (*D*) of the vastus lateralis muscle obtained pre- and post-CT (*n* = 12). Representative ultrasonography images of the vastus lateralis pre- and post-CT (*E*) with the different parameters analyzed (1: fascicle length through extrapolation of the visible part of the fascicle; 2: muscle thickness; 3: echogenicity). All values are expressed as the means ± SD. *P < 0.05, **P < 0.01.

The CSA of the vastus lateralis muscle fibers substantially decreased post-CT (3,830 ± 648 µm^2^ vs. 3,031 ± 508 µm^2^; −21%; *P* < 0.01; Fig. 4*A*), showing muscle atrophy. Muscle architecture was also altered after CT treatment, as shown by a decrease in fascicle length (6.57 ± 0.62 vs. 5.61 ± 0.42 cm; −15%; *P* < 0.05; Fig. 4*B*) and a decreasing trend in muscle thickness (1.85 ± 0.15 vs. 1.71 ± 0.35 cm; −8%; *P* = 0.08; Fig. 4*C*). Echogenicity was not affected, suggesting no change in muscle quality over time (*P* = 0.14; Fig. 4*D*).

## DISCUSSION

This longitudinal clinical study aimed to investigate IMAT content in patients with early breast cancer who were treated with chemotherapy (CT) and explore the subsequent cellular mechanisms. We concomitantly reported a skeletal muscle atrophy and an increase in IMAT content, probably as a consequence of FAP accumulation, as evidenced by the increase in PDGFRα protein levels. Importantly, our study design does not allow us to dissociate the specific effect of the cancer or the chemotherapy treatment, and the development of both skeletal muscle IMAT and atrophy may be the consequence of a delicate combination of physiological stressors, including the disease and its treatment.

### Increase in Vastus Lateralis IMAT Content following Chemotherapy

We showed an increase in IMAT content, as evidenced by a substantial increase in protein levels of key mature adipocyte markers, such as perilipin, adiponectin, and FABP4 (5, 13). If their function is different, they represent commonly used markers of adipocytes (10, 13, 21). It should be noted that these mature adipocytes markers allow us to come to the conclusion of increased IMAT content, but do not allow us to understand the exact role that these proteins might have in this IMAT development. The increase in IMAT content was also supported by the increase in intermuscular lipids content, as shown by the increase in oil red O positive staining quantified between the muscle fibers. Altogether, these results indicate an increase in IMAT content at the cellular level in the vastus lateralis muscle of patients with breast cancer who were treated with CT, and extend previous results from cross-sectional studies comparing patients with healthy controls (15, 16).

We next investigated key adipogenesis markers and found that C/EBPα slightly increased post-CT whereas PPARγ remained unchanged. These results appear paradoxical as given the large increase in mature adipocyte markers post-CT, one could expect to observe an increase in adipogenesis markers. However, by drawing a parallel between the widely studied cellular mechanisms driving muscle atrophy and those of IMAT development, we could also hypothesize that adipogenesis markers might have been upregulated earlier during CT. Indeed, cellular processes triggering muscle atrophy usually return to “normal” expression values when muscle atrophy is established (22–24). In our study, following the completion of 18 wk of CT treatment, the mature adipocytes were well established without any residual upregulation of adipogenesis markers driving adipocyte differentiation. The knowledge that IMAT development mechanisms occur within days of muscle disuse further supports this interpretation (5).

### IMAT Development and Fibro-Adipogenic Progenitor Accumulation: An Unwelcome Combination

Although the implication of FAPs in IMAT development was consensually admitted in various conditions (2, 9, 13, 21), we highlighted FAPs accumulation in patients with breast cancer. Indeed, we established a substantial increase in PDGFRα protein levels post-CT, and FAPs represent the primary cells expressing the cell surface receptor PDGFRα. Thus, our results may indicate that FAPs are implicated in IMAT accumulation in patients with breast cancer undergoing CT but preclinical investigations are necessary to highlight a direct mechanistic link.

Although FAPs essentially promote more fibrotic tissue formation than adipose tissue in various myopathies (10, 11, 25, 26), we did not observe changes in collagen area as well as fibrosis markers such as protein levels of fibronectin and collagen I. Our results suggest that FAPs differentiate into adipose tissue rather than fibrotic tissue in patients with breast cancer who were treated with CT. It will be of interest to determine whether CT directly promotes FAP differentiation into adipocytes, whether an unfavorable cellular microenvironment mediates it, or whether it could be a combination of both (13, 27). Isolation of FAPs from muscle biopsies would allow us to investigate, through in vitro experiments, whether the chemotherapy, the tumor or the combination of both play a direct role in FAP differentiation into adipocytes.

### Are Fibro-Adipogenic Progenitors Responsible for Both IMAT Accumulation and Muscle Atrophy?

We reported substantial muscle atrophy characterized by a decrease in the CSA of vastus lateralis muscle fibers, reaching −21% post-CT. This result is supported by previous studies showing a similar decrease in patients with breast cancer (28) and in preclinical models (29, 30). Undoubtedly, muscle atrophy appears to be a strong maladaptation consecutive to breast cancer treatment, and it negatively influences both treatment tolerance and disease-free survival (1). Using ultrasonography to investigate vastus lateralis muscle architecture, we found a significant reduction in fascicle length, combined with a trend for a decrease in muscle thickness (*P* = 0.08) post-CT. Notably, the decrease in vastus lateralis muscle fiber CSA was comparable with the considerable effect of 60 years of healthy aging (31), whereas the muscle architecture reorganization was comparable with 5 wk of bedrest (32), an extreme muscle disuse model. Both comparisons strongly highlight the profound impact of breast cancer and its chemotherapy treatment on the skeletal muscle apparatus.

In this clinical study, we reported the simultaneous presence of IMAT accumulation and muscle atrophy in patients with breast cancer, as highlighted in many other human skeletal muscle deconditioning models (2, 5, 33, 34). If FAP accumulation plays a critical role in IMAT development, it may also represent an early marker of muscle atrophy. Indeed, FAPs have been recently identified to promote skeletal muscle atrophy in animal models through inflammatory pathways such as IL-6/STAT3 or IL-1β (35, 36). Thus, if we speculate that FAPs represent a crucial initiator of skeletal muscle atrophy in patients with breast cancer, their specific role in the cross talk between skeletal muscle atrophy and IMAT development needs to be extensively studied.

### The Use of Echogenicity to Quantify IMAT Accumulation

Echogenicity, a parameter obtained noninvasively through ultrasonography, has been recommended to assess muscle quality and the indirect presence of IMAT (20). However, despite an increase in IMAT content evidenced in our cellular observations, we did not report any changes in echogenicity. This discrepancy might be explained because echogenicity was validated in the context of rotator cuff tears (20), an injury that can induce “more fat than muscle” according to the Goutallier Staging System for grading fatty infiltration (6). Even if the increase found in mature adipocyte markers is considerable in our experimental setting, our breast cancer patients did not experience the same magnitude of increase. Therefore, echogenicity measured through ultrasonography may not be sensitive enough to detect “small” adaptations into a “large” muscle such as the vastus lateralis, and should be interpreted with caution.

### Conclusions

This longitudinal clinical study showed IMAT development and skeletal muscle atrophy at the cellular level in patients with breast cancer who were treated with chemotherapy. Interestingly, skeletal muscle FAPs likely play a critical role in the skeletal muscle deconditioning observed in patients with breast cancer but further studies are required to decipher the underlying mechanisms and develop therapeutic interventions.

## GRANTS

This work was supported by the Institut de Cancérologie Strasbourg Europe (ICANS) and has been published under the framework of the IdEx Unistra supported by investments in the future program of the French government.

## DISCLOSURES

No conflicts of interest, financial or otherwise, are declared by the authors.

## AUTHOR CONTRIBUTIONS

J.M., E.H., T.J.H., and A.F.P. conceived and designed research; J.M., E.H., L.B., and A.F.P. performed experiments; J.M., E.H., L.B., A.C., L.D., C.P., P.T., R.S., F.F., X.P., T.J.H., and A.F.P. analyzed data; J.M., E.H., L.B., A.C., L.D., C.P., P.T., R.S., F.F., X.P., T.J.H., and A.F.P. interpreted results of experiments; J.M. and E.H. prepared figures; J.M. and A.F.P. drafted manuscript; J.M., E.H., F.F., X.P., T.J.H., and A.F.P. edited and revised manuscript; J.M., E.H., L.B., A.C., L.D., C.P., P.T., R.S., F.F., X.P., T.J.H., and A.F.P. approved final version of manuscript.
